# COVID-19: ramifications of the pandemic on mental health and substance abuse

**DOI:** 10.3389/fpubh.2024.1401734

**Published:** 2024-07-31

**Authors:** Bala Munipalli, Majd Al-Soleiti, Anjali Morris, Teresa Rummans

**Affiliations:** ^1^Division of General Internal Medicine, Mayo Clinic, Jacksonville, FL, United States; ^2^Department of Psychiatry and Psychology, Mayo Clinic, Rochester, MN, United States; ^3^University of Florida, Gainesville, FL, United States

**Keywords:** COVID-19, mental health, substance use, neuroinflammation, neuropsychological effects

## Abstract

**Objective:**

To explore the ramifications of the COVID-19 pandemic on Mental Health and Substance Use.

**Patients and methods:**

Relevant literature examining the correlation between COVID-19 and mental health/substance use was reviewed, and findings were summarized.

**Results:**

Specific mechanisms regarding COVID-19’s effects on the brain are unclear, but preliminary studies and biomarkers have been suggested in the literature. Numerous studies demonstrated COVID-19 has immediate and lingering neuropsychiatric impacts on affected patients. Psychiatric disorders and substance abuse increased during the COVID-19 pandemic due to biological and psychosocial factors, with a significant burden on individuals and societies worldwide, particularly in the United States.

**Conclusion:**

COVID-19 has shown us that underlying causes of mental health and substance abuse problems are more complicated than we have appreciated. Neuroinflammation and psychosocial stresses impact mental health and substance use. These factors need to be explored further for a better understanding and intervention.

## Background

Since the 18th century, viral illnesses have contributed to mental illness and neuropsychiatric disorders directly and indirectly. Complicating the medical aspects of viral epidemics are the mental health, psychosocial, and substance abuse problems. Viral diseases spread rapidly, and those that develop into a pandemic cause significant disruptions to societies and economies worldwide. Following the pandemic of 1918, a significant increase in the number of people diagnosed with schizophrenia occurred (1). Fifty years later, those who had been exposed to the 1918 virus had a significantly higher rate of Parkinson’s disease (2).

Three years after the SARS CoV-1 outbreak in Beijing, there was an increase in alcohol abuse and dependence among specific healthcare workers (men ages 36–50, those with lower educational levels, those with upper-middle level family income, those who worked in units with elevated levels of exposure, and those who had been quarantined) (3). Post-traumatic stress disorder (PTSD) was also the most common psychiatric disorder to arise after the Severe Acute Respiratory Syndrome (SARS) outbreak, with medical staff being significantly affected. Those who had been quarantined and experienced pre-SARS trauma had elevated levels of depressive symptoms 3 years after the outbreak (3, 4).

In 2014, the Ebola virus outbreak was highly infectious and virulent, resulting in high fatality rates and markedly elevated levels of fear and anxiety (5). Jalloh et al. assessed symptoms of anxiety, depression, and post-traumatic stress disorder (PTSD) in the general population in Sierra Leone after over a year of Ebola outbreak response (5). The prevalence of any anxiety-depression symptom was 48%, and of any PTSD symptom, 76% (5). The Middle Eastern Respiratory Syndrome (MERS) in Korea resulted in a 20% mortality rate. 80.2% of the general public reported fear of infection, and 46% reported emotional distress during the outbreak (6).

In March 2020, COVID-19 began to emerge as the latest pandemic. Over the next four years, growing concerns were expressed regarding increasing mental health issues and substance abuse. COVID-19 negatively influenced mental health worldwide due to limited resources for testing and treatment, conflicting messages from health authorities, and uncertain prognoses (7–10). COVID-19 reduced the sense of control individuals experienced, leading to increased addictive symptoms including addictive social media use, and heightened anxiety enhanced by misinformation and fake news provided on social media (11). Reorganization of daily life resulted in increased depression and reduced physical activity in those who had difficulty adapting (11, 12). Interestingly, a collaborative study from four countries (Germany, Italy, Russia, and Spain) demonstrated physical activity could buffer the negative impacts of depression symptoms (13). A study of Italian healthcare workers compared to the general population demonstrated a significant increase in negative mood, worry, restlessness, loneliness, and fatigue (14).

A validated measuring anxiety, the COVID-19 Anxiety Syndrome Scale (C-19ASS) was developed to assess anxiety including maladaptive forms of coping (worry, avoidance, threat monitoring) associated with COVID-19 in the general population in the United States (15). It has been used by numerous other countries (Brazil, China, Greece, Indonesia, the Philippines, Iran, Italy, Saudi Arabia, Turkey, Canada, and the United Kingdom) successfully to demonstrate a significant increase in COVID-19 anxiety syndrome, depression, health anxiety, psychological distress, and functional impairment (9, 10, 15–17).

## Immediate biological impact of COVID-19 on the brain

Since the COVID-19 pandemic started, the SARS-COV-2 virus has been shown to cause multiple neurological symptoms and disorders such as encephalitis, stroke, seizure, delirium, headache, and loss of both senses of smell and taste (18).

Various hypotheses have been proposed to explain how the COVID-19 virus may affect the brain independently of the other effects (19, 20). Two primary biological mechanisms commonly mentioned are primary neuro-invasion of the virus (through destruction of the blood–brain barrier or nerve endings) and secondary systemic changes, both of which may cause various disruptions in the homeostasis of the brain. Multiple studies on animals and human samples have outlined the impact of COVID-19 on the central nervous system (18–21). However, the specific mechanisms remain unclear.

A systematic review by Cosentino et al. analyzed studies that explored neuropathological findings from 438 patients and concluded that a brain inflammatory reaction and hypoxic–ischemic damage rather than neuronal viral load are the mechanisms that underpin neuropsychiatric symptoms caused by SARS-COV-2 infection (22).

A case–control study of 40 participants, published in May 2023 in JAMA Psychiatry, demonstrated that translocator protein total distribution (TSPO VT), which is a marker for gliosis, is elevated in multiple regions of the brain in patients experiencing persistent depressive (low energy, slowed motor speech, anhedonia) and cognitive symptoms associated with COVID-19 illness, compared to controls with COVID-19 but no persistent symptoms (23).

## Lingering biological impact of COVID-19 on the brain

Several studies explored the lingering psychiatric and cognitive complications persisting months after recovery and described in the literature as “Long COVID” or “Post-acute sequelae of COVID-19” (PASC) (24). 57% of COVID-19 patients report at least one long COVID feature 180 days after infection (25).

A scoping review found that Long COVID’s most frequently reported cognitive symptoms were memory (67%) and attentional-executive disturbances (90%). Fatigue was the most commonly reported general symptom (24).

A recent systematic review investigated specific psychiatric symptoms and risk factors of long-term COVID-19 (26). In order of prevalence, the most common symptoms were anxiety, depression, PTSD, poor sleep quality, somatic symptoms, and cognitive deficits. Risk factors associated with these symptoms were being female and having a previous psychiatric diagnosis.

As to how COVID-19 infection relates to patients with pre-existing mental illness, Hovagemyan et al.’s scoping review of 11 studies showed mixed evidence. Six studies noted worse outcomes in the length or severity of symptoms with pre-existing mental illness, while four studies found no correlation between worsening symptoms and psychiatric history (27).

Comparatively, long COVID symptoms occurred more frequently than post-infectious and post-hospitalization syndromes associated with other infections. This was shown in a retrospective cohort study of more than 273 thousand survivors of COVID-19, which compared them to influenza-affected patients (25).

In 2023, an AI-assisted study strengthened evidence suggesting a direct link between COVID-19 and subsequent brain atrophy (28). Magnetic Resonance Imaging (MRI) from patients who recovered from COVID-19 illness was compared against healthy controls. Statistically, significant neocortical brain degeneration was noted, which was increased with greater initial disease severity.

Another study noted MRI abnormalities, particularly multiple white matter lesions, in the majority of patients (71%) experiencing prolonged neuropsychiatric symptoms after COVID-19 infection (29).

## Psychosocial impact of COVID-19 on mental health and substance use

Statistics from the WHO reveal a 13% increase in reported mental health and substance abuse disorders in the decade before 2017 (30). Psychosocial factors attributed to this rise include higher expectations among young adults, social media pressure, lack of boundaries from media exposure, an expectation of instant gratification that leads to anxiety if not satisfied, lack of community involvement, and easy online access to illicit substances.

The pandemic resulted in further isolation, loneliness, illness, grief, food insecurities, job loss, and financial instability, all of which have negatively impacted cognitive abilities and mental health and increased the risk of suicide (31). Intimate partner violence toward women, parental depression, and low self-esteem increased during the pandemic, contributing to adverse effects on children (31).

During the COVID-19 pandemic, peer connections were strained, with <50% of high school students reporting feeling close to their colleagues, resulting in worsening mental health from a lack of social support (32). Adolescent anxiety and depression escalated in 2020 (33), with 47% of parents reporting a negative impact on their children’s mental health by COVID-19 and 17% reporting a “major negative impact” (33). Young adults (ages 18–24) were particularly vulnerable, with 50% reporting anxiety and depression in 2023 (33). Young adults experienced closures of universities and difficulty accessing treatment, compounding poor mental health. Adolescents (and women) experienced more pronounced anxiety and depression, and adolescent females experienced increased feelings of sadness and hopelessness compared to adolescent males (34). A study of 2,869 adolescent participants with mental illness reported a worsening of mental health disorders in 60% of the study population (35). Since the end of the public health emergency, many people have continued to struggle with worsening mental health and experience barriers to care.

From 2019 to 2022, the use of mental health services jumped by almost 40% among US adults with commercial insurance (36). Between April 2020 and February 2023, the percentage of adults reporting symptoms of anxiety and depression rose to 31.5–39.3% from 11% in June 2019 (37). In addition, 53% of individuals experiencing job loss were more likely than those with jobs (30%) to experience anxiety and depression (33). Only 31% of US adults considered their mental health “excellent” in late 2022, compared to 43% in 2002 (36).

Societal disruptions such as the COVID-19 pandemic drove demand on an already taxed system so that some people could not get the support they needed (36). People became more introverted, less creative, less agreeable, and less conscientious, leading to more anxiety and depression (25).

An estimated 45.9 million adults (20% of all adults) in the United States were reported to have a mental illness in 2009 (37). In 2019, prepandemic suicide rates were decreasing. However, Emergency Department (ED) visits in 2020 for mental health crises, including suicide attempts, drug overdoses, and psychosocial stresses (e.g., partner violence, child abuse, and neglect), were significantly higher than in 2019 (38). Self-harm and suicidal ideation increased faster among adolescent females during the pandemic-30% of adolescent females versus 14% of adolescent males considered attempting suicide in 2021, and emergency room visits for suicide attempts increased in adolescent females as the pandemic progressed (38). Major depression episodes in youth rose from 8.66 to 12.63% (25).

The impact of COVID-19 on substance abuse has also been significant. In 2021, 32% of high school students reported the use of alcohol, marijuana, tobacco, and misuse of prescription opioids (33). In 2020, there were 90,000 overdose deaths, of which 50% were associated with fentanyl (up from 70,000 in 2019), the most significant rise in over 20 years (39). Over 100,000 overdose deaths were reported in 2022 (33).

Alcohol-induced death rates increased the fastest among American Indians and Alaska Natives (91.7 per 100,000 in 2021) and were six times higher than Hispanic people, who experienced a rate of 13.6 per 100,000 (40). Black people also experienced significant increases in alcohol-induced deaths during COVID-19, with rates increasing by more than 45% (40). Anxiety and depression and the use of alcohol and illicit drugs were higher among White and Hispanic adolescents and lower among Black adolescents (33). Factors cited included social isolation, economic stress, decreased involvement in community and religious support networks, low income, younger age, lack of access to mental health services, negative news coverage, increasing worry about COVID-19, access to firearms, and reduced sleep.

Drug overdose death rates also increased across all racial and ethnic groups, particularly in people of color, during the pandemic (41). The highest drug overdose death rates in 2021 were among Asian people (56.6 per 100,000), Black people (44.2 per 100,000), and White people (36.8 per 100,000) (33). Drug overdose death rates were also higher among males versus females from 2019 (29.6 vs. 13.7 per 100,000, respectively) to 2021 (45.1 vs. 19.6 per 100,000, respectively) (33).

In Canada, data was collected between December 2020 and June 2021 on the use of cannabis, combustible cigarettes, e-cigarettes, alcohol, and binge drinking in individuals aged 20.4 to 33.6 years (42). Although the majority of participants reported stable substance use, cannabis use increased from 17.5 to 23.1%, e-cigarette use increased from 3.8 to 5.4%, and binge drinking increased by 53.5% (42). Living alone and having a lower education status were associated with increased substance use (42).

Kilian et al. synthesized observational studies published between January 2020 and September 2021 on self-reported changes in alcohol use associated with COVID-19 from European general and clinical populations (43). More individuals indicated a decrease rather than an increase in alcohol use by 3.8% during the pandemic (43). However, among people with pre-existing alcohol use disorder, this drinking pattern intensified during the pandemic, suggesting pre-existing drinking levels impacted pandemic-related alcohol use and a need for ongoing monitoring and support (43).

## Conclusion

COVID-19 has taught us how much we still do not know about the biological and psychosocial aspects of mental illness and substance abuse. Despite the potential limitation of this study not being a systematic review (e.g. PRISMA criteria), there are many important points made that move us forward toward a better understanding of neuropsychiatric problems and their treatment. Both the direct impact of viruses and the secondary indirect biological changes on the brain from COVID-19 producing inflammation, hypoxia, etc. can result in immediate and persistent neuropsychiatric conditions. Consequently, more research is needed focusing on these biological factors such as inflammation, as a cause of many severe mental illnesses including schizophrenia, mania, and severe depression. Better diagnostic tools such as more sophisticated biomarkers, other than sedimentation rate and C Reactive Protein, and more routine use of sensitive brain imaging for early screening and recognition, may allow for the emergence of new treatments for identified causes of neuropsychiatric symptoms. Additionally, psychosocial factors are often ignored until it is too late to reverse the impact on mental illness and substance abuse. Early screening and recognition could result in better clinical outcomes. An interdisciplinary approach and treatment with integrated care models, including added support in schools and the workforce to identify those struggling with mental illness or substance abuse early are essential to addressing psychosocial factors. Public health education and community engagement through raised awareness, enhanced screening, and early detection could reduce stigma and result in better policies and more support services. Recognizing and addressing these issues that we have learned from the ramifications of COVID-19 will help us improve the care of those with neuropsychiatric illnesses.

## Author contributions

BM: Project administration, Resources, Supervision, Validation, Visualization, Writing – original draft, Writing – review & editing, Methodology, Investigation, Formal analysis, Data curation, Conceptualization. MA-S: Writing – review & editing, Writing – original draft, Visualization, Methodology, Investigation, Formal analysis, Data curation, Conceptualization. AM: Resources, Writing – review & editing, Writing – original draft. TR: Conceptualization, Data curation, Formal analysis, Funding acquisition, Investigation, Methodology, Project administration, Supervision, Validation, Visualization, Writing – original draft, Writing – review & editing.
