# Erratum: COVID-19: ramifications of the pandemic on mental health and substance abuse

**DOI:** 10.3389/fpubh.2024.1477635

**Published:** 2024-09-16

**Authors:** Frontiers Production Office

**Affiliations:** Frontiers Media SA, Lausanne, Switzerland

**Keywords:** COVID-19, mental health, substance use, neuroinflammation, neuropsychological effects

Due to an error, the incorrect manuscript was used for typesetting. Corrections have been made to the text and references, as outlined below.

A correction has been made to the section Background, paragraph 4:

“In March 2020, COVID-19 began to emerge as the latest pandemic. Over the next four years, growing concerns were expressed regarding increasing mental health issues and substance abuse. COVID-19 negatively influenced mental health worldwide due to limited resources for testing and treatment, conflicting messages from health authorities, and uncertain prognoses (7–10). COVID-19 reduced the sense of control individuals experienced, leading to increased addictive symptoms including addictive social media use, and heightened anxiety enhanced by misinformation and fake news provided on social media (11). Reorganization of daily life resulted in increased depression and reduced physical activity in those who had difficulty adapting (11, 12). Interestingly, a collaborative study from four countries (Germany, Italy, Russia, and Spain) demonstrated physical activity could buffer the negative impacts of depression symptoms (13). A study of Italian healthcare workers compared to the general population demonstrated a significant increase in negative mood, worry, restlessness, loneliness, and fatigue (14).

A validated measuring anxiety, the COVID-19 Anxiety Syndrome Scale (C-19ASS) was developed to assess anxiety including maladaptive forms of coping (worry, avoidance, threat monitoring) associated with COVID-19 in the general population in the United States (15). It has been used by numerous other countries (Brazil, China, Greece, Indonesia, the Philippines, Iran, Italy, Saudi Arabia, Turkey, Canada, and the United Kingdom) successfully to demonstrate a significant increase in COVID-19 anxiety syndrome, depression, health anxiety, psychological distress, and functional impairment (9, 10, 15–17).”

A correction has also been made to the section Conclusion:

“COVID-19 has taught us how much we still do not know about the biological and psychosocial aspects of mental illness and substance abuse. Despite the potential limitation of this study not being a systematic review (e.g. PRISMA criteria), there are many important points made that move us forward toward a better understanding of neuropsychiatric problems and their treatment. Both the direct impact of viruses and the secondary indirect biological changes on the brain from COVID-19 producing inflammation, hypoxia, etc. can result in immediate and persistent neuropsychiatric conditions. Consequently, more research is needed focusing on these biological factors such as inflammation, as a cause of many severe mental illnesses including schizophrenia, mania, and severe depression. Better diagnostic tools such as more sophisticated biomarkers, other than sedimentation rate and C Reactive Protein, and more routine use of sensitive brain imaging for early screening and recognition, may allow for the emergence of new treatments for identified causes of neuropsychiatric symptoms.

Additionally, psychosocial factors are often ignored until it is too late to reverse the impact on mental illness and substance abuse. Early screening and recognition could result in better clinical outcomes. An interdisciplinary approach and treatment with integrated care models, including added support in schools and the workforce to identify those struggling with mental illness or substance abuse early are essential to addressing psychosocial factors. Public health education and community engagement through raised awareness, enhanced screening, and early detection could reduce stigma and result in better policies and more support services. Recognizing and addressing these issues that we have learned from the ramifications of COVID-19 will help us improve the care of those with neuropsychiatric illnesses.

The following references were added:

7. Pfefferbaum B, North CS. Mental health and the Covid-19 pandemic. *N Engl J Med*. (2020) 383:510–2. doi: 10.1056/NEJMp2008017

8. Nikčević AV, Marino C, Kolubinski DC, Leach D, Spada MM. Modelling the contribution of the Big Five personality traits, health anxiety, and COVID-19 psychological distress to generalised anxiety and depressive symptoms during the COVID-19 pandemic. *J Affect Disord*. (2021) 279:578–84. doi: https://doi.org/10.1016/j.jad.2020.10.053

9. Akbari M, Seydavi M, Babaeifard M, Firoozabadi MA, Nikčević AV, Spada MM. Psychometric properties and psychological correlates of the COVID-19 Anxiety Syndrome Scale: a comprehensive systematic review and meta-analysis. *Clin Psychol Psychother*. (2023) 30:931–49. doi: 

10. Seth R, Madathil SA, Siqueira WL, McNally M, Quiñonez CR, Glogauer M, et al. Validity and reliability of the COVID-19 Anxiety Syndrome Scale in Canadian dentists. *Clin Psychol Psychother*. (2023) 30:1349–56. doi: https://doi.org/10.1002/cpp.2877

11. Brailovskaia J, Margraf J. The relationship between burden caused by coronavirus (Covid-19), addictive social media use, sense of control and anxiety. *Comput Human Behav*. (2021) 119:1067206. doi: https://doi.org/10.1016/j.chb.2021.106720

12. Brailovskaia J, Miragall M, Margraf J, Herrero R, Baños RM. The relationship between social media use, anxiety and burden caused by coronavirus (COVID-19) in Spain. *Curr Psychol*. (2022) 41:7441–7. doi: https://doi.org/10.1007/s12144-021-01802-8

13. Brailovskaia J, Cosci F, Mansueto G, Miragall M, Herrero R, Baños RM, et al. The association between depression symptoms, psychological burden caused by Covid-19 and physical activity: an investigation in Germany, Italy, Russia, and Spain. *Psychiatry Res*. (2021) 295:113596. doi: https://doi.org/10.1016/j.psychres.2020.113596

14. Mansueto G, Lopes FL, Grassi L, Cosci F. Impact of COVID-19 outbreak on Italian healthcare workers versus general population: results from an online survey. *Clin Psychol Psychothe*r. (2021) 28:1334–45. doi: https://doi.org/10.1002/cpp.2644

15. Nikčević AV, Spada MM. The COVID-19 anxiety syndrome scale: development and psychometric properties. *Psychiatry Res*. (2020) 292:113322. doi: https://doi.org/10.1016/j.psychres.2020.11332

16. Alhakami A, Salem V, Alateeq D, Nikčević AV, Marci T, Palmieri S, et al. The Arab COVID-19 Anxiety Syndrome Scale (C-19ASS): COVID-19 anxiety syndrome and psychological symptoms in the Saudi Arabian population. *Clin Psychol Psychother*. (2023) 30:1083–94. doi: https://doi.org/10.1002/cpp.2860

17. Mansueto G, Palmieri S, Marino C, Caselli G, Sassaroli S, Ruggiero GM, et al. The Italian COVID-19 Anxiety Syndrome Scale: investigation of the COVID-19 anxiety syndrome and its association with psychological symptoms in an Italian population. *Clin Psychol Psychother*. (2022) 29:1972–90. doi: https://doi.org/10.1002/cpp.2767

The publisher apologizes for this mistake. The original version of this article has been updated.
